# Allergic diseases in children with attention deficit hyperactivity disorder: a systematic review and meta-analysis

**DOI:** 10.1186/s12888-017-1281-7

**Published:** 2017-03-31

**Authors:** Celine Miyazaki, Momoko Koyama, Erika Ota, Toshiyuki Swa, Linda B. Mlunde, Rachel M. Amiya, Yoshiyuki Tachibana, Kiwako Yamamoto-Hanada, Rintaro Mori

**Affiliations:** 1 Department of Health Policy, National Center for Child Health and Development, 10-1-2 Okura, Setagaya-ku, Tokyo, 157-8535 Japan; 2 Department of Community and Global Health, Graduate School of Medicine, The University of Tokyo, 7-3-1 Hongo, Bunkyo, Tokyo, 113-8654 Japan; 3 Global Health Nursing, Graduate School of Nursing Science, St. Luke’s International University, 10-1 Akashicho, Chuo-ku, Tokyo, 104-0044 Japan; 4 Graduate School of Human Sciences, Osaka University, 1-1 Yamadaoka Suita, Osaka Prefecture, 565-0871 Japan; 5 Department of Family Nursing, Graduate School of Medicine, The University of Tokyo, 7-3-1 Hongo, Bunkyo, Tokyo, 113-8654 Japan; 6 Department of Psychosocial Medicine, National Center for Child Health and Development, 10-1-2 Okura, Setagaya-ku, Tokyo, 157-8535 Japan; 7 Department of Medical Specialties, National Center for Child Health and Development, 10-1-2 Okura, Setagaya-ku, Tokyo, 157-8535 Japan

**Keywords:** Allergic conjunctivitis, Allergic disease, Allergic rhinitis, Asthma, Atopic dermatitis, Attention deficit hyper disorder, Coexisting condition, Food allergy, Meta-analysis

## Abstract

**Background:**

Reports of frequent manifestation of allergic diseases in children with attention deficit hyperactivity disorder (ADHD) have been the subject of mounting clinical interest. However, evidence supporting the association between ADHD and allergies is inconsistent and has yet to be systematically reviewed. The objective of this study was to compile and assess available studies on the association between ADHD and allergic diseases in children.

**Methods:**

A comprehensive search using MEDLINE, EMBASE, the Cochrane library, and CINAHL databases was completed in 23 November 2015. The inclusion criteria for studies were that the research assessed allergic diseases in children, 18 years of age and younger, with a diagnosis of ADHD and that a distinct comparison group was incorporated. Any comparative studies, encompassing both randomized controlled trials and observational studies, were considered for inclusion. Two review authors independently assessed the quality of the selected studies by the use of validated assessment tools, performed data extraction and conducted meta-analysis according to Cochrane Collaboration guidelines.

**Results:**

Five eligible studies were included in this systematic review. Of these studies, three were case-control and two were cross sectional studies. A majority of information from the five studies was classified as having low or unclear risk of bias. The meta-analysis showed an association between children with ADHD and asthma compared with the control groups (OR: 1.80, 95% CI: 1.57 - 2.07; five studies, low quality of evidence), but did not indicate an association between food allergy and ADHD (OR: 1.13, 95% CI: 0.88 - 1.47; three studies very low quality of evidence). The odds of experiencing allergic rhinitis, atopic dermatitis, and allergic conjunctivitis were slightly higher in children with ADHD compared with control groups, though a substantial statistical heterogeneity was notable in the overall effect estimates.

**Conclusions:**

The findings from this review and meta-analysis show that children with ADHD are more likely to have asthma, allergic rhinitis, atopic dermatitis, and allergic conjunctivitis than their counterparts. Interventions including strategies for managing allergies in children with ADHD would be beneficial.

**Electronic supplementary material:**

The online version of this article (doi:10.1186/s12888-017-1281-7) contains supplementary material, which is available to authorized users.

## Background

The association between attention deficit hyper disorder (ADHD) and allergic diseases, whether rooted in comorbidity or causality, has been a source of public and clinical concern since the 1980s [1]. The most recent estimate of worldwide ADHD prevalence in children and adolescents is reported at 7.1% [2]. ADHD is often recognized in early-school children with persistent patterns of inattention and hyperactivity-impulsivity that interfere in functioning, social development or both and the symptoms usually persist to adulthood [3]. There are few therapy options (e.g., behavioral therapy or cognitive training) for children with ADHD, but the effectiveness varies depending on the individual because of the complex array of factors (e.g., genetic and environmental conditions) that intertwine with the behavioral interpretation during their development [3, 4]. While a cure for ADHD has not yet been fully achieved, the available clinical treatment for children with ADHD, including adolescents and adults, is largely medication-based (e.g., methylphenidate, amphetamine or atomoxetine adrenergic agonistics drugs), and the prescribed medications are to improve certain aspects of attention span and hyperactivity behavior [5].

Whether ADHD is related to hypersensitivity or not has not been fully discerned, but numerous reports on allergies in children with ADHD have created a growing concern among healthcare providers. Several studies have documented cases of allergic manifestations (e.g., atopic dermatitis or asthma) in response to stimulant and non-stimulant drug treatments for ADHD, but the explanation for the allergic disease manifestations remained ambiguous [6–8]. In the aspect of the neurophysiological mechanism, some studies have suggested that the relationship between the immune response and the central nervous system (CNS) may predispose some children to autism, impulsive behavior or ADHD [9–14]. However, the proposed interrelated mechanism of the elevated proinflammatory cytokines reactivity in the brain found to be triggered by allergic response could does not adequately explain the different types of allergy manifested in the children with ADHD [15, 16]. With environmental factors (e.g., family or school setting and social distress) and other coexisting conditions (e.g., metabolic abnormalities, sleep disorders and epilepsy) being suggested to influence the severity of ADHD symptoms, it is necessary to gain a better understanding of the relationship between allergy and ADHD from both biological and epidemiological perspectives toward providing the most desirable care possible [17, 18].

Several epidemiological studies have reported that children with ADHD have a high risk of developing allergic diseases, such as asthma and atopic dermatitis, while other studies have reported no evidence of a link between allergy and ADHD [19–22]. Given such conflicting evidence on the association, it is suspected that a sizeable proportion of the ADHD population experiencing comorbidities with various allergic diseases may have been overlooked. Moreover, the resulting lack of clear recommendations in this area have meant that the optimal benefit of tailored interventions and supportive care may not reach those children in need. The objective of this systematic review was thus to compile and analyze the best available evidence on whether rates of allergic diseases are significantly higher in children with ADHD and to identify the specific types of allergic diseases to which such children may be prone.

## Methods

### Search strategy

This review was conducted in accordance with the Cochrane Handbook for Systematic Reviews and the Preferred Reporting Items for Systematic reviews and Meta-Analyses (PRISMA) statement, and the PRIMSA checklist is provided in an additional file (see Additional file 1) [23, 24]. A search strategy was developed, with an information specialist, to meet the review question ‘Is there an association between ADHD and allergies?’ and the comprehensive search was completed on 23 November 2015 by the use of MEDLINE, EMBASE, the Cochrane library and CINAHL databases. To ensure the search was as comprehensive as possible, subject terms were exploded so as to include narrower terms, regardless their wide range word expressions in the text search. The search terms included ‘child development disorders, pervasive’, ‘mental disorders diagnosed in childhood’, ‘attention deficit disorder’, ‘learning disorder’, ‘autism’ and ‘hypersensitivity’ (see Additional file 2 for search strategy details). There was no date, language or types of publication restriction imposed on the search. The retrieved references and the articles were managed by the use of EndNote version X6 software (Copyright © 2012 Thomson Reuters).

### Study selection and quality assessment

Five authors reviewed titles and abstracts of all potentially eligible articles retrieved from the databases. Three authors in one group and two authors in another group independently screened all the titles and abstracts from the bibliographic list retrieved, and the reference lists of the retrieved article were additionally hand-searched where necessary. After irrelevant studies were removed in this initial stage, two authors collected the full-text of the potentially relevant studies and independently examined the report content for determining eligibility based on our pre-specification criteria for including and excluding the studies. When studies referred to previously published protocols or results reported elsewhere, those referenced studies were retrieved and examined as well. After eligible studies had been selected, the studies were then coded by the first author’s last name and publication year as their identification in this systematic review. Any disagreement was resolved by discussion with other authors or consultation with other experts in this subject.

The eligibility criteria for including studies for this review were developed by following the general methods for Cochrane reviews and the Non-randomised Studies Methods Group (NRSMG) of the Cochrane Collaboration guidelines [23]. The participants that met this review eligibility were children and adolescents who were 18 years of age or younger and were clinically diagnosed or identified through psychiatric assessment as having ADHD, and with the diagnosis of allergic diseases or immune hypersensitivity. Mixed-gender studies, single-gender studies and studies of ADHD-diagnosed children with or without record of receiving treatment were eligible for inclusion. The definition of ADHD was determined by the established Diagnostic and Statistical Manual of Mental Disorders, Fourth Edition, Text Revision (DSM-IV-TR) and DSM-5, and the National Institute for Health and Care Excellence (NICE) clinical guideline 72 on ADHD [25, 26]. In addition, studies that referenced use of structured diagnostic interviews and validated screening tools (e.g., Conners Parent Rating Scale (CPRS) or Child Behavior Checklist (CBCL) scales) in evaluating ADHD symptoms were considered eligible for inclusion. In regard to the diagnosis of allergic diseases, studies were considered for inclusion if the diagnosis was done under physical examination or laboratory test, or otherwise met the clinical criteria for the diagnosis of allergy (e.g., International Classification of Disease, Injuries, and Causes of Death [ICD]). There was no restriction placed on the types of study design included. Randomized controlled trials and all comparative studies (e.g., prospective, longitudinal, retrospective, case-control or cross sectional studies) with a comparison group of children without clinical indication of developmental disorders were all considered eligible for this review.

The exclusion criteria were studies that reported pre-specified comorbidities within other categories of developmental disorders (e.g., autism spectrum disorders or specific learning disabilities) or neurological disorders, such as epilepsy. Finally, studies that included only adult population, studies without distinguishable comparison groups, studies using animal models, systematic reviews, case series reports, and articles that did not provide original data were considered irrelevant for inclusion.

To appraise the validity of the studies, two authors used risk of bias tools to perform quality and risk of bias assessment for all the eligible studies independently. The Cochrane risk-of-bias assessment tool was used to assess clinical trial studies, and the Risk of Bias Assessment Tool for Non-randomized Studies (RoBANS), equivalent to that of the Cochrane risk-of-bias assessment tool, was used to assess the observational studies [27]. There were six main domains for potential bias evaluation in RoBANS and each domain was categorized as ‘low risk’, ‘high risk’, or ‘unclear risk’. The methodological quality of the primary studies were examined and judged as either low, high or unclear risk of bias in relation to the assessment domains. To enhance consistency of judgment and decision making between the review authors, we additionally referred to the guidance from the Cochrane Risk of Bias Assessment Tool: for Non-Randomized Studies of Interventions (ACROBAT-NRSI) that described the types of bias for selection of participants, confounding, measurement of interventions, measurement of outcomes, missing data and selection of reported results for clarification [28]. After the risk of bias assessment was completed, both authors independently extracted the data from the eligible primary studies and recorded the extracted data to a modified data collection form with items of required information listed. The information items were consistent with our pre-specified criteria and were formatted to seek for the characteristic of the studies, setting, definition of the ADHD population, types of allergy and results of any outcome measures. The extracted data were then transferred to the Review Manager (RevMan) 5.3 software for meta-analysis [29].

### Data synthesis

Studies with similar characteristic were combined for meta-analysis. When relevant outcome data were available for synthesis, they were entered into RevMan 5.3 software for pair-wise comparison. The Mantel-Haenszel method was used for assessing dichotomous outcomes, whereas for continuous outcomes, the inverse variance method would be used. The relative effect measures were calculated by using odds ratio (OR) statistics based on the reporting from the studies, and the relative effect estimate was assigned with a 95% confidence interval (CI) and a *p*-value cut-off point of 0.05. Since diagnosis tools used to assess ADHD were highly varied among the studies due to cultural and clinical practice differences across countries, the true effect would be most likely varied from one study to the next. For this reason, a random-effects model assumption was used. If the number of included studies was very small or the study designs were too diverse, both random-effects and fixed-effects models were used to test the trend of the estimated effect as well. To determine heterogeneity, the chi-squared method was used with a cut-off point of 0.10 to determine statistical significance. The I^2^ statistic was used to calculate consistency for the combined studies to test the impact of heterogeneity in the meta-analysis. When the reason for substantial heterogeneity was unclear, subgroup analysis was performed as a means of investigating heterogeneous results and identifying whether the difference between groups could have interaction to the effect magnitude. The sensitivity analysis was performed to examine whether the overall estimate in the meta-analysis was affected by studies that were different in their sampling approach (e.g., population-based designs as opposed to hospital, school, or community-based designs) or not.

### Grading of evidence

To evaluate the quality of available data on the association between ADHD and allergy diseases, the Grading of Recommendations Assessment, Development and Evaluation (GRADE), supported by guidelines outlined in the GRADE handbook was used [30]. The strength of the evidence were divided into four quality grades: high, moderate, low, and very low, according to confidence in the estimate lying close to the true effect [31]. Determinations of quality were based on five factors: methodological limitations creating risk of bias within the study, inconsistency of results, indirectness of evidence, imprecision of results, and publication bias. Quality ratings for observational studies began from ‘low’, with the possibility of upgrading if further research would be likely to provide confidence in the estimate effect or no threats to validity and evidence of a dose-response or exposure-response gradient were found. Studies were downgraded as ‘very low’ quality if they contained uncertainty about the directness of results or contained unsystematic observations [32].

## Results

**Fig. 1:**
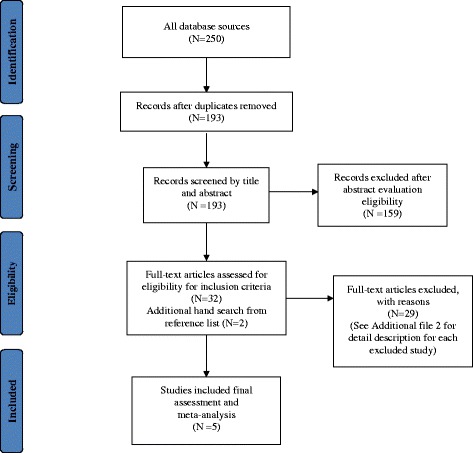
Flow diagram for search process and selection of studies

The comprehensive search identified a total of 261 studies from the databases (Fig. 1). After removing duplicates, 240 studies were screened by title and abstracts for potential inclusion. Out of 240 studies, 21 studies were selected for full-text examination. Of those 21 studies, 15 studies did not meet the entry criteria and were excluded with reasons provided (e.g., twin studies, studies with no specified comparison group or behavior assessments were made according to the allergic severity and not by ADHD clinical diagnoses; see Additional file 3 for details on excluded studies with reasons for exclusion). There were multiple reports of the same population identified and they were considered as one study for meta-analysis, but those multiple reports that showed to have overlapping results, only the study with the most relevant and high-quality data among the reports were selected for inclusion. There were no eligible RCT studies identified related to ADHD and allergies. After the final selection process, five studies, all observational in their study design, were found to meet the inclusion criteria. Of the five eligible studies, one was a population-based case-control study [33], two were case-control studies [34, 35], and two were cross sectional studies [36, 37] (Table 1). These studies were conducted in Germany, Korea, the United Kingdom (UK), Taiwan, and Thailand with varied data sources. Among these studies, one study [35] collected data from hospital patients and one study [36] collected data from the elementary schools. The remaining three studies collected data from national medical research databases: one study [33] used National Health Insurance Research Database (NHIR), one study [34] used UK General Practice Research Database (GPRD) and one study [37] used German Health Interview and Examination Survey for Children and Adolescents. Among the five included studies, only one study [34] indicated that children with ADHD had at least one record of a methylphenidate prescription within 12 moths after the date of first diagnosis. The rest of the four studies did not include background information regarding prescribed medications.

**Table 1:** Summary of characteristics of included studies

Study ID	Location	Study design (Period)	Participants (Gender, male%)	Age range in years (Mean ± SD)	Definition of ADHD symptoms	Allergic diseases types	Outcome measurements
Chen 2013 [33]	Taiwan	Case-control (1996-2010)	*N* = 39, 880 *ADHD: n = 5811; Tic alone: n = 1816; ADHD and Tic: n = 349; general population n = 31,904* (76%; 77.4%)	ADHD: (15 ± 81) Control: (17 ± 17)	ADHD was diagnosed according to ICD-9-CM code by psychiatrists.	• Asthma • Allergic rhinitis • Atopic dermatitis • Allergic conjunctivitis	Allergic diseases were classified according to ICD-9-CM codes by internists/pediatrician.
Hak 2013 [34]	UK	Nested case-control (1996-2006)	*N* = 4, 420 *ADHD: n = 884; control: n = 3536* (Restricted to male only)	4 to14 years	Based on medical records of first-time diagnosis of ADHD (at least one recorded prescription of ADHD drug)	• Asthma • Allergic rhinitis • Atopic dermatitis • Any atopic disorder • Unspecific allergies • Food allergy • Cow’s milk intolerance	Diagnoses of atopic disorders, asthma were done based on codes recorded on medical histories
Kwon 2014 [36]	Korea	Cross sectional (not specified: data collected all year long)	*N* = 4113 *ADHD: n = 549; control: n = 3564* (75.2%; 47.9%)	7 to 8 years	Diagnosed according to DSM-IV criteria, together with epidemiological questionnaires Computerized Attention Deficit-Hyperactivity Disorder Diagnostic System, the abbreviated Conner’s Parent Rating Scale (CPRS), and DuPaul’s ADHD Rating Scales.	• Asthma • Allergic rhinitis • Atopic dermatitis • Allergic conjunctivitis • Food allergy Drug allergy	Measured by questionnaires, International Study of Asthma and Allergies in Children (ISAAC), for asthmatic symptoms/skin rashes/sneezing
Romanos 2010 [37]	Germany	Retrospective cross sectional (2003-2006)	*N* = 13, 318 *ADHD: n = 653; control: n = 12,665* (79.6%; 48.4%)	3 to 17 years	Medical examination according to ICD-10 criteria and standardized parental interviews.	• Asthma • Allergic rhinitis • Atopic eczema	Self reported and medical examination
Suwan 2011 [35]	Thailand	Case-control (January-November 2010)	*N* = 80 *ADHD n = 40; control n = 40* (77.5%; 77.5%)	5 to15 years	Diagnosed according to DSM-IV criteria by pediatrician	• Asthma • Allergic rhinitis • Eczema • Allergic conjunctivitis • Urticaria • Food allergy	Evaluated by history and physical examination and skin prick tests

Of the five included studies, four studies [33–35, 37] were judged to have an unclear risk of bias and only one study [36] was judged to have a high risk of bias for selective outcome reporting based on RoBANS criteria (see Additional file 4 for the risk of bias assessment). With regard to the selection of participants, four studies were at unclear risk of bias [33, 34, 36, 37] whereas one study was at low risk of bias [35]. As for potential bias due to confounding, all studies were judged to have a low risk of bias. Regarding to measurement of exposure, four studies were at low risk of bias [33–35, 37] and one study was at unclear risk of bias [36]. In the blinding of outcome assessment domain, one study was at low risk of bias [37] but the rest of the four studies were at unclear risk of bias. All the studies were at low risk of bias in terms of bias caused by incomplete of outcome data. As for selective outcome reporting, two studies were at unclear risk of bias [33, 34], one study was at high risk of bias [36], and two studies were at low risk of bias [35, 37]. Further details on the supporting judgments for risk of bias assessments are presented in Additional file 4.

There was a total of 61,811 children involved in the five included studies and 38,324 (62%) of the children were males. Of the 61,811 children, 7937 (13%) were diagnosed with ADHD. The types of allergic diseases reported in these studies were asthma, allergic rhinitis, atopic dermatitis, and allergic conjunctivitis. Other allergies such as food allergy, drug allergy, urticatia, any atopic disorder or unspecified allergies were also reported in some studies (see Additional file 5 for a summary of results on allergic diseases in children with ADHD). In performing the meta-analysis, studies were stratified by their sample selection approach as part of the sensitivity analysis. Two studies [33, 37] were of population-based design and they were grouped as (nationwide studies). As for the remaining three studies [34–36], the data were collected from selected schools or hospitals; wherefore, it was grouped as (institutional-based studies).

### Asthma

**Fig. 2:**
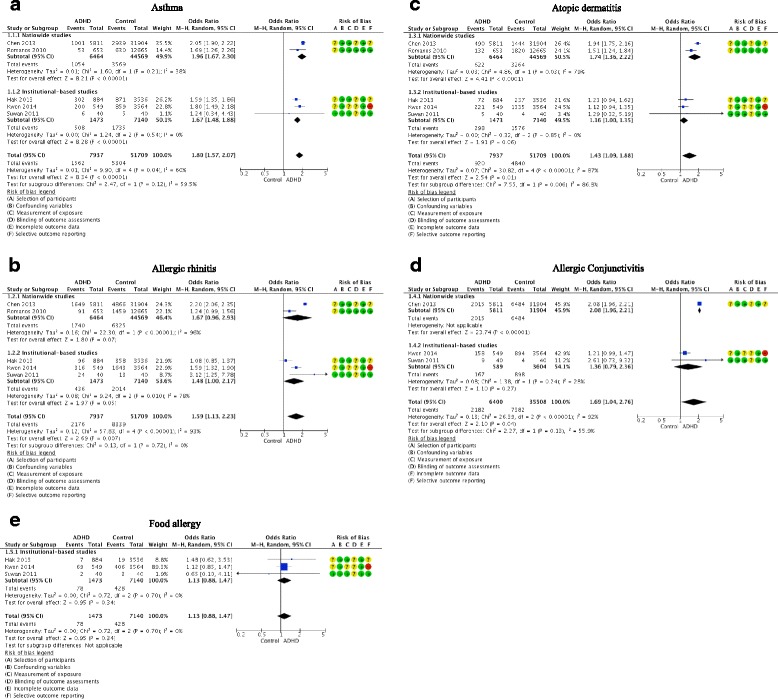
Forest plots of allergic diseases in children with ADHD and in control children. **a** Asthma. **b** Allergic. **c** Atopic dermatitis. **d** Allergic conjunctivitis. **e** Food allergy.

Five studies examined the risk of asthma in children with ADHD compared to children without ADHD (Fig. 2a). The meta-analysis showed that children with ADHD were nearly twice as likely to have asthma compared with those in the control groups (OR: 1.80, 95% CI: 1.57 - 2.07, I^2^ = 60%; five studies, *n* = 59,646 children). The pooled estimate from the studies showed a statistically significant difference between the two groups, and the large overall estimate indicated an association between asthma and ADHD. The heterogeneity across the studies was found to be statistically non-significant but a moderate inconsistency was detected. To explore the influence of nationwide studies versus institutional-based studies on the effect estimate, subgroup analysis was conducted. There was no significant difference found between the nationwide studies [33, 37] and the institutional-based studies [34–36].

### Allergic rhinitis

Five studies evaluated the risk of allergic rhinitis in children with ADHD compared to those without ADHD (Fig. 2b). The result from the combined studies showed that a higher proportion of children in the ADHD groups had allergic rhinitis compared to the control groups (OR: 1.59, 95% CI: 1.13 - 2.23, I^2^ = 93%; five studies, *n* = 59,646 children). This suggests that children with ADHD experienced 59% greater odds of having allergic rhinitis relative to the children without ADHD; however, substantial evidence of heterogeneity and inconsistency were detected among the studies. Regarding to subgroup analysis, there was no significant difference indicated between the nationwide studies [33, 37] and the institutional-based studies [34–36].

### Atopic dermatitis

Five studies assessed the association between atopic dermatitis and ADHD in children (Fig. 2c). From the pooled estimated across the studies, the relative effect indicated a significant difference between the ADHD and the control groups, with cosiderable heterogeneity and inconsistency found across the studies (OR: 1.43, 95% CI: 1.09 -1.88, I^2^ = 87%, five studies, *n* = 59,646). In the subgroup analysis, an apparent difference was detected (*p* = 0.006). The nationwide studies [33, 37] showed that the children in the ADHD groups had higher odds of atopic dermatitis relative to the control groups (OR: 1.74, 95% CI: 1.36 - 2.22, I^2^ = 79%; two studies, *n* = 51,033 children), and there was a substantial heterogeneity and inconsistency found between the studies. From the institutional-based studies [34–36], the odds of atopic dermatitis appeared to be only slightly higher in the ADHD groups compared with the control groups (OR: 1.16, 95% CI: 1.00 - 1.35, I^2^ = 0%; three studies, *n* = 8613 children), and the effect estimate did not reach statistical significance as the confidence interval crossed the line of null effect.

### Allergic conjunctivitis

Three studies provided data for allergic conjunctivitis in children with ADHD compared with children without ADHD (Fig. 2d). The pooled estimate from these studies showed that a higher proportion of children in the ADHD groups experienced allergic conjunctivitis compared with the control groups (OR: 1.69, 95% CI: 1.04 - 2.76; I^2^ = 92%, three studies, *n* = 41,908); however, there was notable heterogeneity and a substantial inconsistency detected across the studies. Differences between the subgroups, meanwhile were not statistically significant.

### Food allergy

Three studies examined the association between ADHD and food allergy; all of which were institutional-based studies (Fig. 2e). The meta-analysis showed no significant difference between the ADHD groups and the control groups, as the confidence interval of the odds ratio estimate included the the null value (OR: 1.13, 95% CI: 0.88 - 1.47, I^2^ = 0%; three studies, *n* = 8613). There was no implication of inconsistency and heterogeneity between the studies in the pooled result.

### Quality of evidence

The quality of evidence for asthma associated with ADHD in children was considered low quality, largely due to information from one study [36] contributing a serious risk of bias, thus posing a major threats to the validity of information derived from the studies (Table 2). As for allergic rhinitis, atopic dermatitis, allergic conjunctivitis and food allergy, the quality of the evidence was downgraded from low to very low for serious risk of bias and inconsistency between the included studies. Details on the specific factors, such as overall risk of bias, inconsistency, indirectness, imprecision and publication bias, that influenced the evidence quality are described in the summary of evidence quality table, based on the GRADE approach (see Additional file 6). Publication bias assessment was considered to be inappropriate since only five studies were identified in this review. As a test for funnel plot symmetry should ideally include at least ten studies, the test for publication bias was not adequately powered to determine whether this was truly an issue in our analyses.

**Table 2:** Summary of findings

Allergy diseases in children with ADHD					
Population: Children with ADHD Setting: Germany, Korea, Taiwan, Thailand, UK Intervention: none (observation of the difference in risk of allergy diseases) Comparison: Children without ADHD Outcome: Allergic diseases					
Allergy diseases	Anticipated absolute effects* (95% CI)		Relative effect (95% CI)	№ of participants (studies)	Quality of the evidence (GRADE)
	Control group risk (Assumed risk)	ADHD group risk (Corresponding risk)			
Asthma	125 per 1000	205 per 1000 (183 to 228)	OR 1.80 (1.57 to 2.07)	59,646 (5 studies)	⨁⨁◯◯ LOW ^a,h^
a) Nationwide studies	a) 71 per 1000	a) 130 per 1000 (113 to 149)	a) OR 1.96 (1.67 to 2.30)	a) 51,033 (2 studies)	a) ⨁◯◯◯ VERY LOW ^b,h^
b) Institutional-based studies	b) 241 per 1000	b) 347 per 1000 (320 to 374)	b) OR 1.67 (1.48 to 1.88)	b) 8613 (3 studies)	b) ⨁◯◯◯ VERY LOW ^c.h^
Allergic rhinitis	153 per 1000	222 per 1000 (169 to 286)	OR 1.59 (1.13 to 2.23)	59,646 (5 studies) a)	⨁◯◯◯ VERY LOW ^a,d,e,h^
a) Nationwide studies	a) 134 per 1000	a) 205 per 1000 (129 to 312)	a) OR 1.67 (0.96 to 2.93)	b) 51,033 (2 studies)	a) ⨁◯◯◯ VERY LOW ^b,d,e,h^
b) Institutional-based studies	b) 325 per 1000	b) 416 per 1000 (325 to 511)	b) OR 1.48 (1.00 to 2.17)	c) 8613 (3 studies)	b) ⨁◯◯◯ VERY LOW ^c,d,e,h^
Atopic dermatitis	100 per 1000	137 per 1000 (108 to 173)	OR 1.43 (1.09 to 1.88)	59,646 (5 studies)	⨁◯◯◯ VERY LOW ^a,d,h^
a) Nationwide studies	a) 94 per 1000	a) 154 per 1000 (124 to 188)	a) OR 1.74 (1.36 to 2.22)	a) 51,033 (2 studies)	a) ⨁◯◯◯ VERY LOW ^b,d,h^
b) Institutional-based studies	b) 100 per 1000	b) 114 per 1000 (100 to 130)	b) OR 1.16 (1.00 to 1.35)	b) 8613 (3 studies)	b) ⨁◯◯◯ VERY LOW ^c,h^
Allergic Conjunctivitis	203 per 1000	301 per 1000 (210 to 413)	OR 1.69 (1.04 to 2.76)	41,908 (3 studies)	⨁◯◯◯ VERY LOW ^c,d,e,h^
a) Nationwide studies	a) 203 per 1000	a) 347 per 1000 (333 to 360)	a) OR 2.08 (1.96 to 2.21)	a) 37,715 (1 study)	a) ⨁⨁◯◯ LOW ^f^
b) Institutional-based studies	b) 175 per 1000	b) 224 per 1000 (144 to 334)	b) OR 1.36 (0.79 to 2.36)	b) 4193 (2 studies)	b) ⨁◯◯◯ VERY LOW ^g,d,e,h^
Food allergy Institutional-based studies	75 per 1000	84 per 1000 (67 to 106)	OR 1.13 (0.88 to 1.47)	8613 (3 studies)	⨁◯◯◯ VERY LOW ^c,e,h^

*The risk in the ADHD group (and its 95% confidence interval) is based on the assumed risk in the comparison group and the relative effect of the exposure (and its 95% CI)

*CI* confidence interval, *OR* odds ratio

GRADE Working Group grades of evidence (level of evidence of grading for observational studies)

Very low: Observational studies with uncertainty about the directness of results or unsystematic observations

Low: Observational studies with no threats to validity

Moderate: Observational studies with no threats to validity and evidence of a dose-response or exposure-response gradient

High: Observational studies with no threats to validity yielding very large effects

^a^ Four of the studies had limitation on the selection of participants and blinding of outcomes assessments by their study designs but one study had unclear risk of bias in the measurement of exposure and one study indicated high risk of bias on the outcome reporting which lowered the quality of the observational evidence

^b^ The proportion of information was from two studies indicated with limitation on selection of participants and blinding by their study designs but the unclear risk of selective reporting which lowered the quality of the observational evidence

^c^ The proportion of information was from two studies indicated limitation on selection of participants and blinding of outcome assessments by their study designs but one study had high risk of outcome reporting, which lowered the quality of the observational evidence

^d^ There is an indication of significant inconsistency (I^2^ > 80%)

^e^ Information were from high heterogeneity and small sample size with a wide confidence interval

^f^ The information is based on one study, which had limitation on the selection of participants, blinding of outcomes assessments and selective outcome reporting by the study design

^g^ The information from two studies that had limitation on selection of participants, measurement of exposure, blinding of outcome assessment by their study designs but one with high risk of bias on selective outcome reporting which lowered the quality of the observational evidence

^h^ The possibility of publication bias is not disregarded but it was not considered to downgrade the quality of the observational evidence

## Discussion

This systematic review was designed to compile and present the best available evidence for the association between children with ADHD and allergic diseases in children - a relationship hitherto not yet addressed in a randomized controlled trial. Our meta-analysis of the identified observational studies indicated that children with ADHD have an 80% increased odds of asthma compared with children without ADHD. The high asthma rate in children with ADHD could potentially be linked to the recent discovery of genetic association, in which several studies suggested that a gene polymorphism of dopamine receptor D5 (DRD5), a form of the dopamine D1-like receptor, is associated with a ADHD behavior subtype and that the expression of DRD5 is found in both the mammalian brain and peripheral blood leukocytes [38–40]. According to these reports, DRD5 may also engage in some part of the immunological regulation process of T helper 17 cell (Th17) differentiation, which is extensively involved in asthma development [41]. Available literature thus seemingly supports the possible comorbidity of ADHD and asthma, but the correlation between these multiple mechanisms has not yet been clarified.

For allergic rhinitis, atopic dermatitis, and allergic conjunctivitis, the estimated relative odds were slightly higher in children with ADHD than in children without ADHD. The low statistical power most likely resulted from the statistical heterogeneity and the methodological diversity of the included studies, with a further potential confounder in factors that were not measured (e.g., climate, pollution or microbial agents). Since allergic rhinitis and conjunctivitis are triggered by seasonal or household allergens, insufficient covariate adjustment from the primary studies could yield heterogeneity in the meta-analyses; for example, cross sectional data may have been collected during a period when particular allergens are naturally more prevalent [42]. Although the overall odds ratio for atopic dermatitis was deemed small, the nationwide studies from the subgroup analysis showed a significantly higher rate of atopic dermatitis in children with ADHD than in the control groups, and interestingly, the estimate was similar to the result for asthma. In line with that observation, a recent study of atopic dermatitis reported that there was a high frequency of filaggrin (*FLG*) null alleles detected in children of European origin with both atopic dermatitis and asthma phenotype [43, 44]. Perhaps it would be advantageous to more closely examine in future studies the comorbidity of atopic allergies and asthma in children with ADHD, particularly, whether the allergic march, in terms of the progression of various allergic conditions with age, and genetic penetrance could be a potential link.

Regarding to food allergy, there was no significant difference found between children with ADHD and children in the control groups. One underlying reason for this could be the complexity of the Immunoglobulin E (IgE) immune response to food allergens in the gastrointestinal tract falling between the tolerance and sensitization mechanisms [45]. In line with the present findings, a previous systematic review similarly found no evidence for an association between serum-IgE levels and ADHD symptoms [20]. Although the complex genetic associations between ADHD and immunological regulations in the CNS have been emphasized in this review, considering the onset and the vast array of neuropsychiatric disorder outcomes, neuronal signaling and immune regulation thus represented only a small part of the pathogenesis [10, 46].

The overall quality of evidence for an association between allergic diseases and ADHD children was found to be low for asthma and very low for atopic dermatitis, allergic rhinitis, allergic conjunctivitis, and food allergy. The differences of study designs and studies with risk of bias were largely attributed to the downgrading of the quality. Perhaps more case-control studies with standardized protocols could allow for better effect estimation and minimize the risk of bias posed in observational studies, thus improving the validity of meta-analyses in future.

This review provides a summary of the overall level of evidence for an association between ADHD and allergies. However, certain limitations should be considered when interpreting this review. The evidence from these studies pertains only to the association between ADHD and allergies under the speculation of possibly coexisting conditions and not on the onset and the causative pathways between the two conditions. Furthermore, this review was not able to obtain information on the stimulant or non-stimulant medication used in the ADHD population, except for in one study [34], where the author mentioned that the children with ADHD had at least one prescription record of methylphenidate. Since that study excluded those who did not used methylphenidate from the assessment, a comparison group was not available for analysis, and the evidence of an association between stimulant used for ADHD and allergies remains lacking. In several of the identified studies [33, 34, 37], the control group of children was selected based on the absence of ADHD medical records with one study excluded female children; therefore, potential ADHD cases in the control group were not considered. With a small sample size in one study, an overestimation of power for the pooled standardized mean could likely influence the magnitude of the observed relationships between ADHD and allergic diseases. To enhance the quality of evidence, the presence of potential confounding factors in terms of cognitive ability, family environment (e.g., parents’ mental health, parenting attitudes), and other potential coexisting mental diseases (e.g., anxiety disorder, major depressive disorder, autism spectrum disorder) should be elucidated. Additionally, medication sensitivity, bacterial or fungal infection and preterm birth should also be adequately identified and adjusted for in analyses.

## Conclusions

This systematic review showed that children with ADHD had elevated rates of asthma compared to the children without ADHD. An association between food allergy and ADHD, meanwhile, was not evident based on the meta-analysis. The odds of allergic rhinitis, atopic dermatitis, and allergic conjunctivitis in children with ADHD was found to be slightly higher than in children without ADHD, though the overall effect size was affected by substantial heterogeneity across the studies. Interventions incorporating strategies that focus on allergic disease management and collaborative care for children with ADHD might be beneficial to explore.

## Additional files

Additional file 1: PRISMA checklist. (DOCX 38 kb)

Additional file 2: Search strategy. (DOCX 20 kb)

Additional file 3: Excluded studies with reasons for exclusion. (DOCX 145 kb)

Additional file 4: Risk of bias assessment within studies. (DOCX 134 kb)

Additional file 5: Summary of results on allergic diseases in children with ADHD. (DOCX 22 kb)

Additional file 6: Summary of evidence quality based on GRADE approach. (DOCX 130 kb)

## Abbreviations

ACROBAT-NRSI: Cochrane risk of bias assessment tool: for non-randomized studies of Interventions
ADHD: Attention deficit hyperactivity disorder
CBCL: Child behavior checklist
CI: Confidence interval
CNS: Central nervous system
CPRS: Conners parent rating scale
DAT: Dopamine transporter
DMS-IV-TR: Diagnostic and statistical manual of mental disorders, fourth edition, text revision
DRD5: Dopamine receptor D5
DSM-5: Diagnostic and statistical manual of mental disorders, fifth edition
*FLG*: Gene encoding filaggrin
GRADE: The grading of recommendations assessment, development and evaluation
ICD: The international classification of diseases, injuries, and causes of death
IgE: Immunoglobulin E
ISAAC: International study of asthma and allergies in children
NICE: National Institute for Health and Care Excellence
NRSMG: Non-randomised Studies Methods Group
OR: Odds ratio
PRISMA: Preferred reporting items for systematic reviews and meta-analyses
RoBANS: Risk of bias assessment tool for non-randomized studies
Th17: T helper 17 cell
