# Supplementary material for: Allergic diseases in children with attention deficit hyperactivity disorder: a systematic review and meta-analysis
Source: BMC Psychiatry. 2017 Mar 31;17:120. doi: 10.1186/s12888-017-1281-7 (PMC5374627; doi:10.1186/s12888-017-1281-7)
Supplement: Supplementary file 1 — PRISMA checklist. (DOCX 38 kb) [file 12888_2017_1281_MOESM1_ESM.docx]

**Additional file 1: PRISMA checklist**

Entitle: **Allergic diseases in children with attention deficit hyperactivity disorder: a systematic review and meta-analysis**

Authors: Celine Miyazaki, MSc, PhD; Koyama Momoko, MSc; Erika Ota, RNM, PhD; Toshiyuki Swa, MA; Linda Beatrice Mlunde, M.D., MSc, PhD; Rachel Marie Amiya, MSc, PhD; Yoshiyuki Tachibana, M.D., PhD; Kiwako Yamamoto-Hanada, M.D; Rintaro Mori, M.D., PhD

*PRISMA Checklist template was from* (www.prisma-statement.org)*:* Moher D, Liberati A, Tetzlaff J, Altman DG, The PRISMA Group (2009). Preferred Reporting Items for Systematic Reviews and Meta-Analyses: The PRISMA Statement. Ann Intern Med. 2009;151:264-269.

| **Section/topic** | **#** | | **Checklist item** | **Reported on page #** |
| --- | --- | --- | --- | --- |
| **TITLE** | | | |  |
| Title | | 1 | Identify the report as a systematic review, meta-analysis, or both. | p.1  ‘Allergic diseases in children with attention deficit hyperactivity disorder: a systematic review and meta-analysis’ |
| **ABSTRACT** | | | |  |
| Structured summary | 2 | | Provide a structured summary including, as applicable: background; objectives; data sources; study eligibility criteria, participants, and interventions; study appraisal and synthesis methods; results; limitations; conclusions and implications of key findings; systematic review registration number. | p.3  Background: ‘Reports of frequent manifestation of allergic diseases in children with attention deficit hyperactivity disorder (ADHD) have been the subject of mounting clinical interest.’  Objective: ‘The objective of this study was to compile and assess available studies on the association between ADHD and allergic diseases in children.’  Methods: ‘A comprehensive search using MEDLINE, EMBASE, the Cochrane library, and CINAHL databases, etc.’  Eligibility criteria, participants and intervention: ‘...the research assessed allergic diseases in children, 18 years of age and younger, with a diagnosis of ADHD and that a distinct comparison group was incorporated. Any comparative studies, encompassing both randomized controlled trials and observational studies, were considered for inclusion.’  Study appraisal and synthesis methods: ‘...the quality of the selected studies by the use of validated assessment tools, performed data extraction and conducted meta-analysis according to Cochrane Collaboration guidelines.’  Results: ‘Five eligible studies were included in this systematic review. Of these studies, three were case-control and two were cross sectional studies and so forth.’  Conclusion: ‘Interventions including strategies for managing allergies in children with ADHD would be beneficial.’ |
| **INTRODUCTION** | | | |  |
| Rationale | 3 | | Describe the rationale for the review in the context of what is already known. | p.7  ‘Given such conflicting evidence on the association, it is suspected that a sizeable proportion of the ADHD population experiencing comorbidities with various allergic diseases may have been overlooked.’ |
| Objectives | 4 | | Provide an explicit statement of questions being addressed with reference to participants, interventions, comparisons, outcomes, and study design (PICOS). | p.7  ‘The objective of this systematic review was thus to compile and analyze the best available evidence on whether rates of allergic diseases are significantly higher in children with ADHD and to identify the specific types of allergic diseases to which such children may be prone.’ |
| **METHODS** | | | |  |
| Protocol and registration | 5 | | Indicate if a review protocol exists, if and where it can be accessed (e.g., Web address), and, if available, provide registration information including registration number. | p. 7;  The review protocol was constructed by following the *Cochrane Handbook for Systematic Reviews of Interventions* and the reporting of this review follows the PRISMA guideline*.* |
| Eligibility criteria | 6 | | Specify study characteristics (e.g., PICOS, length of follow-up) and report characteristics (e.g., years considered, language, publication status) used as criteria for eligibility, giving rationale. | p.8  ‘To ensure the search was as comprehensive as possible, subject terms were exploded so as to include narrower term, regardless their wide range word expressions, in the free-text search.’  ‘There was no date, language or types of publication restriction imposed on the search.’ |
| Information sources | 7 | | Describe all information sources (e.g., databases with dates of coverage, contact with study authors to identify additional studies) in the search and date last searched. | p.8  ‘...the comprehensive search was completed on 23 November 2015, etc.’ ‘When studies referred to previously published protocols or indicated results reported elsewhere, those referenced studies were retrieved and examined for as well.’ |
| Search | 8 | | Present full electronic search strategy for at least one database, including any limits used, such that it could be repeated. | p.8  ‘... by the use of MEDLINE, EMBASE, the Cochrane library and CINAHL databases.’ (See Additional file 2 for search strategy details)’ |
| Study selection | 9 | | State the process for selecting studies (i.e., screening, eligibility, included in systematic review, and, if applicable, included in the meta-analysis). | p.8 to 9  The process for selecting studies is described in the *Study selection and quality assessment* section. (See Figure 1 for the selection process.) |
| Data collection process | 10 | | Describe method of data extraction from reports (e.g., piloted forms, independently, in duplicate) and any processes for obtaining and confirming data from investigators. | p.10  ‘After the risk of bias assessment was completed, two authors independently extracted the data from the eligible primary studies and recorded them to a modified data collection form, with items of information to be sought listed, as recommended in the Cochrane Handbook.’  ‘The extracted data were then transferred to the Review Manager (RevMan) 5.3 software for meta-analysis’ |
| Data items | 11 | | List and define all variables for which data were sought (e.g., PICOS, funding sources) and any assumptions and simplifications made. | p.10  ‘The information items were consistent with our pre-specified criteria such as, characteristic of the studies, setting, definition of the ADHD population, types of allergy and results of any outcome measures.’ |
| Risk of bias in individual studies | 12 | | Describe methods used for assessing risk of bias of individual studies (including specification of whether this was done at the study or outcome level), and how this information is to be used in any data synthesis. | p.10  ‘To appraise the validity of the studies, two authors used risk of bias tools to perform quality and risk of bias assessment for all the eligible studies independently. The Cochrane risk-of-bias assessment tool was used to assess clinical trial studies, and the Risk of Bias Assessment Tool for Non-randomized Studies (RoBANS), equivalent to that of the Cochrane risk-of-bias assessment tool, was used to assess the observational studies.’ (See Additional file 4 for the risk of bias assessment criteria and results)’ |
| Summary measures | 13 | | State the principal summary measures (e.g., risk ratio, difference in means). | p.11  ‘The relative effect measures were calculated by using odds ratio (OR) statistics based on the reporting from the studies, and the relative effect estimate was assigned with a 95% confidence interval (CI) and a p-value cut-off point of 0.05.’ |
| Synthesis of results | 14 | | Describe the methods of handling data and combining results of studies, if done, including measures of consistency (e.g., I^2^) for each meta-analysis. | p.11  ‘...a random-effects model assumption was used. If the number of included studies was very small or the study designs were too diverse, both random-effect and fixed-effect models were used to test the trend of the estimated effect as well. To determine heterogeneity, the chi-squared method was used with a cut-off point of 0.10 to determine statistical significance. The I2 statistic was used to calculate consistency for the combined studies to test the impact of heterogeneity in the meta-analysis.’ |
| Risk of bias across studies | 15 | | Specify any assessment of risk of bias that may affect the cumulative evidence (e.g., publication bias, selective reporting within studies). | p.11  ‘To evaluate the quality of available data on the association between ADHD and allergy diseases, The Grading of Recommendations Assessment, Development and Evaluation (GRADE), supported by guidelines outlined in the GRADE handbook was used.’ (See Additional file 6 for GRADE tables) |
| Additional analyses | 16 | | Describe methods of additional analyses (e.g., sensitivity or subgroup analyses, meta-regression), if done, indicating which were pre-specified. | p.11  ‘When the reason for substantial heterogeneity was unclear, subgroup analysis was performed as a means of investigating heterogeneous results and identifying whether the difference between groups could have interaction to the effect magnitude.’ |
| **RESULTS** | | | |  |
| Study selection | 17 | | Give numbers of studies screened, assessed for eligibility, and included in the review, with reasons for exclusions at each stage, ideally with a flow diagram. | p.12  (See Figure 1 for flow diagram)  (See Additional file 3 for reasons for exclusions) |
| Study characteristics | 18 | | For each study, present characteristics for which data were extracted (e.g., study size, PICOS, follow-up period) and provide the citations. | p.12  (See Table 1) |
| Risk of bias within studies | 19 | | Present data on risk of bias of each study and, if available, any outcome level assessment (see item 12). | p.13  ‘Of the five included studies, four studies [33-35, 37] assessed as at unclear risk of bias and only one study [36] as at high risk of bias for selective outcome reporting based on RoBANS criteria (see Additional file 4).’ |
| Results of individual studies | 20 | | For all outcomes considered (benefits or harms), present, for each study: (a) simple summary data for each intervention group (b) effect estimates and confidence intervals, ideally with a forest plot. | p.13 to16  (See Additional file 5 summary of data for each group)  (See Figure 2 for effect estimates and forest plot) |
| Synthesis of results | 21 | | Present results of each meta-analysis done, including confidence intervals and measures of consistency. | p.14 to16  Meta-analysis results are described in *Asthma, Allergic rhinitis, Atopic dermatitis, Allergic conjunctivitis, and Food Allergy* section. |
| Risk of bias across studies | 22 | | Present results of any assessment of risk of bias across studies (see Item 15). | p.16  Assessment results are described in *Quality of evidence* section.  (See Additional file 6) |
| Additional analysis | 23 | | Give results of additional analyses, if done (e.g., sensitivity or subgroup analyses, meta-regression [see Item 16]). | p.14 to 16  Subgroup analysis results are described in *Asthma, Allergic rhinitis, Atopic dermatitis, Allergic conjunctivitis, and Food Allergy* section.  (See Figure 2) |
| **DISCUSSION** | | | |  |
| Summary of evidence | 24 | | Summarize the main findings including the strength of evidence for each main outcome; consider their relevance to key groups (e.g., healthcare providers, users, and policy makers). | p.17  The main findings including the strength of evidence for each main outcome are described in *Discussion* section.  (See Table 2 for the summary of findings) |
| Limitations | 25 | | Discuss limitations at study and outcome level (e.g., risk of bias), and at review-level (e.g., incomplete retrieval of identified research, reporting bias). | p. 19  The limitations are described in *Discussion* section. |
| Conclusions | 26 | | Provide a general interpretation of the results in the context of other evidence, and implications for future research. | p. 20  See *Conclusion* section. |
| **FUNDING** | | | |  |
| Funding | 27 | | Describe sources of funding for the systematic review and other support (e.g., supply of data); role of funders for the systematic review. | p. 22  See *Funding* section. |
