# Supplementary material for: Allergic diseases in children with attention deficit hyperactivity disorder: a systematic review and meta-analysis
Source: BMC Psychiatry. 2017 Mar 31;17:120. doi: 10.1186/s12888-017-1281-7 (PMC5374627; doi:10.1186/s12888-017-1281-7)
Supplement: Supplementary file 2 — Search strategy. (DOCX 20 kb) [file 12888_2017_1281_MOESM2_ESM.docx]

**Additional file 2: Search strategy**

| **MEDLINE** | |
| --- | --- |
| Nov 23, 2015 | |
| **ID** | **Search terms** |
| 1 | exp *Child Development Disorders, Pervasive |
| 2 | exp *"Attention Deficit and Disruptive Behavior Disorders" |
| 3 | exp *Learning Disorders |
| 4 | or/1-3 |
| 5 | exp *Hypersensitivity |
| 6 | 4 and 5 |
| 7 | remove duplicates from 6 |
| 8 | limit 7 to humans |
| 9 | limit 8 to (comment or congresses or editorial or historical article or interactive tutorial or introductory journal article or lectures or legal cases or letter or news or newspaper article or overall or patient education handout) |
| 10 | 8 not 9 |

| **EMBASE** | |
| --- | --- |
| Nov 23, 2015 | |
| **No.** | **Search terms** |
| #1 | 'autism'/exp/mj |
| #2 | 'attention deficit disorder'/mj |
| #3 | 'learning disorder'/exp/mj |
| #4 | #1 OR #2 OR #3 |
| #5 | 'hypersensitivity'/exp/mj |
| #6 | #4 AND #5 |
| #7 | #6 AND [humans]/lim AND [embase]/lim NOT [medline]/lim |

| **The Cochrane Library** | | |
| --- | --- | --- |
| Nov 23, 2015 | | |
| **Cochrane DARE** | | |
| **ID** | | **Search terms** |
| #1 | | MeSH descriptor: [Child Development Disorders, Pervasive] explode all trees |
| #2 | | MeSH descriptor: [Attention Deficit and Disruptive Behavior Disorders] explode all trees |
| #3 | | MeSH descriptor: [Learning Disorders] explode all trees |
| #4 | | #1 or #2 or #3 |
| #5 | | MeSH descriptor: [Hypersensitivity] explode all trees |
| #6 | | #4 and #5 |
| **Cochrane CDSR** | | |
| **ID** | **Search terms** | |
| #1 | MeSH descriptor: [Child Development Disorders, Pervasive] explode all trees | |
| #2 | MeSH descriptor: [Attention Deficit and Disruptive Behavior Disorders] explode all trees | |
| #3 | MeSH descriptor: [Learning Disorders] explode all trees | |
| #4 | #1 or #2 or #3 | |
| #5 | MeSH descriptor: [Hypersensitivity] explode all trees | |
| #6 | #4 and #5 | |
| **Cochrane CCTR** | | |
| **ID** | **Search terms** | |
| #1 | MeSH descriptor: [Child Development Disorders, Pervasive] explode all trees | |
| #2 | MeSH descriptor: [Attention Deficit and Disruptive Behavior Disorders] explode all trees | |
| #3 | MeSH descriptor: [Learning Disorders] explode all trees | |
| #4 | #1 or #2 or #3 | |
| #5 | MeSH descriptor: [Hypersensitivity] explode all trees | |
| #6 | #4 and #5 | |

| **CINAHL** | |
| --- | --- |
| Nov 23, 2015 | |
| **ID** | **Search terms** |
| S1 | (MM "Child Development Disorders, Pervasive+") |
| S2 | (MM "Attention Deficit Hyperactivity Disorder") |
| S3 | (MM "Learning Disorders+") |
| S4 | S1 or S2 or S3 |
| S5 | (MM "Hypersensitivity+") |
| S6 | S4 and S5 |
| S7 | S6 Limiters- Research Article; Exclude MEDLINE records |
