# Supplementary material for: Allergic diseases in children with attention deficit hyperactivity disorder: a systematic review and meta-analysis
Source: BMC Psychiatry. 2017 Mar 31;17:120. doi: 10.1186/s12888-017-1281-7 (PMC5374627; doi:10.1186/s12888-017-1281-7)
Supplement: Supplementary file 3 — Excluded studies with reasons for exclusion. (DOCX 145 kb) [file 12888_2017_1281_MOESM3_ESM.docx]

**Additional file 3: Excluded studies with reasons for exclusion**

| Study ID and reference | Reason for exclusion | Country |
| --- | --- | --- |
| Biederman 1994  *Biederman J, Milberger S, Faraone SV, Guite J, Warburton R. Associations between childhood asthma and ADHD: issues of psychiatric comorbidity and familiality. J Am Acad Child Adolesc Psychiatry. 1994;33(6):842-8.* | It was a familial correlation study of ADHD proband and relatives and the history of asthma prevalence. The control group data was only used to compare for baseline difference but not for the asthma association. | USA |
| Boris 2004  *Boris M, Goldblatt A. Pollen exposure as a cause for the deterioration of neurobehavioral function in children with autism and attention deficit hyperactive disorder: nasal pollen challenge. Journal of Nutritional & Environmental Medicine. 2004;14(1):47-54.* | It was a controlled trial but the comparison was between ASD group and ADHD group of children. | USA |
| Brawley 2004  *Brawley A, Silverman B, Kearney S, Guanzon D, Owens M, Bennett H, et al. Allergic rhinitis in children with attention-deficit/hyperactivity disorder. Ann Allergy Asthma Immunol. 2004;92(6):663-7.* | Case study with no independent control group for comparison. | USA |
| Chang 2013  *Chang HY, Seo J-H, Kim HY, Kwon J-W, Kim B-J, Kim HB, et al. Allergic diseases in preschoolers are associated with psychological and behavioural problems. Allergy, asthma & immunology research. 2013;5(5):315-21.* | The symptoms of mental health and behavior were assessed with ISAAC and CBCL questionnaire but the participants were not confirmed with clinical diagnosis of ADHD. | Korea |
| Chen 2014  *Chen M-H, Su T-P, Chen Y-S, Hsu J-W, Huang K-L, Chang W-H, et al. Is atopy in early childhood a risk factor for ADHD and ASD? a longitudinal study. Journal of psychosomatic research. 2014;77(4):316-21.* | Multiple report of Chen 2013. The same Taiwan National Health Insurance Research Database from 1996 to 2010 was used. A high possibility of overlapping the population selection. | Taiwan |
| Chou 2013  *Chou P-H, Lin C-C, Lin C-H, Loh E-W, Chan C-H, Lan T-H. Prevalence of allergic rhinitis in patients with attention-deficit/hyperactivity disorder: a population-based study. Eur Child Adolesc Psychiatry. 2013;22(5):301-7.* | A report study on the same population selection (Taiwan National Health Insurance Research Database) during the time frame of Chen 2013. Overlapping population selection is highly suspected. | Taiwan |
| Ehlayel 2014  *Ehlayel MSS, Bener A, Bener HZ. Association between asthma and attention-deficit hyperactivity disorders in children: Potential risk factors. Allergy: European Journal of Allergy and Clinical Immunology. 2014;69:231.* | The selection of case group was of children with comorbidity of asthma and ADHD that compared to control group of health children. The study was to evaluate vitamin D deficiency. | Qatar |
| Garg 2014  Garg N, Silverberg JI. Association between childhood allergic disease, psychological comorbidity, and injury requiring medical attention. Annals of allergy, asthma & immunology: official publication of the American College of Allergy, Asthma, & Immunology. 2014;112(6):525-32. | The survey analysis did not clarify whether the children were diagnosed with ADHD under standardized criteria or had medical record of the diagnosis. | USA |
| Genuneit 2014  *Genuneit J, Braig S, Brandt S, Wabitsch M, Florath I, Brenner H, et al. Infant atopic eczema and subsequent attention-deficit/hyperactivity disorder-a prospective birth cohort study. Pediatric allergy and immunology: official publication of the European Society of Pediatric Allergy and Immunology. 2014;25(1):51-6.* | The population that overlapped the population selection from the national database of (2003-2006) of age from 3-17 in the included study, Romanos 2010. The recruited population is between 2000-2001 but they study was a follow up study of the participants at age 1, 2, 3, 4, 6, 8, and 11. | Germany |
| Glemitz 2015  *Glemnitz M, Wölfer W, Krauel K, Bonnekoh B, Röttger U, Flechtner H, et al. Atopic dermatitis and attention deficit hyperactivity disorder: Altered granule transport mechanisms pave the road to atopy. Experimental Dermatology. 2015;24(3):E1.* | The data published in abstract format without sufficient description and information for further evaluation. The group of children with Atopic dermatitis and ADHD were excluded from their analysis. | Germany |
| Grizenko 2015  *Grizenko N, Osmanlliu E, Fortier ME, Joober R. Increased risk of asthma in children with ADHD: Role of prematurity and maternal stress during pregnancy. Journal of the Canadian Academy of Child and Adolescent Psychiatry. 2015;24(2):109-15.* | There was no data on the independent control group of non-ADHD children for comparison. This study assessed on the cofounding factors of pregnancy, delivery and perinatal complications within the selected group of ADHD children. | Canada |
| Heilbrum 2015  *Heilbrun LP, Palmer RF, Jaen CR, Svoboda MD, Miller CS, Perkins J. Maternal chemical and drug intolerances: Potential risk factors for autism and attention deficit hyperactivity disorder (ADHD). Journal of the American Board of Family Medicine. 2015;28(4):461-70.* | Allergic diseases were not described or defined. | USA |
| Holmberg 2015  *Holmberg K, Lundholm C, Anckarsäter H, Larsson H, Almqvist C. Impact of asthma medication and familial factors on the association between childhood asthma and attention-deficit/hyperactivity disorder: A combined twin- and register-based study. Clinical and Experimental Allergy. 2015;45(5):964-73.* | A cross-sectional study of twins and without an independent comparable control group. | Sweden |
| Lee 2014  *Lee YS, Kim SH, You JH, Baek HT, Na C, Kim BN, et al. Attention deficit hyperactivity disorder like behavioral problems and parenting stress in pediatric allergic rhinitis. Psychiatry Investigation. 2014;11(3):266-71.* | ADHD symptoms were evaluated but the diagnosis was not confirmed medically. | Korea |
| McGee 1993  *McGee R, Stanton WR, Sears MR. Allergic disorders and attention deficit disorder in children. J Abnorm Child Psychol. 1993;21(1):79-88.* | Allergic diseases were exposed at 9 years old but did not measure their behavior for baseline comparison. They only measure the behavior problems at age 13. | New Zealand |
| McQuaid 2008  *McQuaid EL, Weiss-Laxer N, Kopel SJ, Mitchell DK, Nasau JH, Wamboldt MZ, et al. Pediatric asthma and problems in attention, concentration, and impulsivity: disruption of the family management system. Families, Systems & Health: The Journal of Collaborative Family HealthCare. 2008;26(1):16-29.* | The evaluation is on symptoms of ADHD, which did not confirm how many of the children had been diagnosed with ADHD clinically previously. There was no independent comparison control group. | USA |
| Mogensen 2011  *Mogensen N, Larsson H, Lundholm C, Almqvist C. Association between childhood asthma and ADHD symptoms in adolescence--a prospective population-based twin study. Allergy. 2011;66(9):1224-30.* | Twins study with no specified independent comparison control group. | Sweden |
| Roth 1991  *Roth N, Beyreiss J, Schlenzka K, Beyer H. Coincidence of attention deficit disorder and atopic disorders in children: empirical findings and hypothetical background. J Abnorm Child Psychol. 1991;19(1):1-13.* | ADD symptoms were measured as the outcome but did not indicate weather the children had been diagnosed with ADD previously in both the atopic eczema and the control group. There were no clear diagnoses results on how many fall into the diagnostic criteria of DSM-III | Germany |
| Schmitt 2009  *Schmitt J, Romanos M, Schmitt NM, Meurer M, Kirch W. Atopic eczema and attention-deficit/hyperactivity disorder in a population-based sample of children and adolescents. Jama. 2009;301(7):724-6.* | A study used the national database (2003 and 2004) that overlapped with the population selection from same database of (2003-2006) Romanos 2010. | Germany |
| Schmitt 2010  *Schmitt J, Apfelbacher C, Chen C-M, Romanos M, Sausenthaler S, Koletzko S, et al. Infant-onset eczema in relation to mental health problems at age 10 years: results from a prospective birth cohort study (German Infant Nutrition Intervention plus). Journal of Allergy and Clinical Immunology. 2010;125(2):404-10.* | The study surveyed the emotion and conduct behavior using SDQ but the results did not fall specifically in the clinical diagnostic criteria of ADHD. | Germany |
| Schmitt 2011  *Schmitt J, Chen CM, Apfelbacher C, Romanos M, Lehmann I, Herbarth O, et al. Infant eczema, infant sleeping problems, and mental health at 10 years of age: the prospective birth cohort study LISAplus. Allergy. 2011;66(3):404-11.* | Multiple report of Schmitt 2009 study. The study surveyed the emotion and conduct behavior using SDQ but the results did not fall specifically in the clinical diagnostic criteria of ADHD. | Germany |
| Shyu 2012  *Shyu C-S, Lin H-K, Lin C-H, Fu L-S. Prevalence of attention-deficit/hyperactivity disorder in patients with pediatric allergic disorders: a nationwide, population-based study. J Microbiol Immunol Infect. 2012;45(3):237-42.* | A multiple report study on the same population selection (national database 2005) during the time frame of (1996-2010) Chen 2013. Overlapping population selection to the included study of Chen 2013. | Taiwan |
| Sleath 2014  *Sleath B, Sulzer SH, Carpenter DM, Slota C, Gillette C, Sayner R, et al. Communication about ADHD and its treatment during pediatric asthma visits. Community mental health journal. 2014;50(2):185-92.* | The purpose of this study was to examine: (a) whether providers discuss key areas recommended by the AAP ADHD guidelines, (b) the extent to which providers discuss, educate, and ask questions about ADHD medications during pediatric visits, (c) the extent to which children and parents ask questions about ADHD and its treatment, and (d) the extent to which the provider asks for and includes child input into the ADHD treatment regimen. | USA |
| Son 2015  *Son JH, Lee YS, Han J, Bahn GH. Comorbidity of allergic diseases among patients with ADHD and Tourette's disorder: A nationwide population-based study. ADHD Attention Deficit and Hyperactivity Disorders. 2015;7:S71.* | The data published in abstract format without sufficient description and information for evaluation. The nationwide data could include the population from the study of Kwon 2014 on the co-morbidity of ADHD in Korea. | Korea |
| Tsai 2011  *Tsai M-C, Lin H-K, Lin C-H, Fu L-S. Prevalence of attention deficit/hyperactivity disorder in pediatric allergic rhinitis: a nationwide population-based study. Allergy Asthma Proc. 2011;32(6):41-6.* | A multiple report study on the same population selection (national database 2005) during the time frame of (1996-2010) Chen 2013. Overlapping population selection to the included study of Chen 2013 | Taiwan |
| Tsai 2012  *Tsai JD, Lue KH. Association between allergic diseases and attention-deficit-hyperactivity disorder in childhood: A population-based study. Developmental Medicine and Child Neurology. 2012;54((Tsai J.-D.; Lue K.-H.) Chung Shan Medical Hospital, Taichung City, Taiwan):69.* | A poster session report and the method indicated a multiple report study on the same population selection (national database 2001-2002) during the time frame of (1996-2010) Chen 2013. Overlapping population selection to the study of Chen 2013 | Taiwan |
| Wolfer 2014  *Wölfer W, Krauel K, Bonnekoh B, Röttger U, Flechtner H, Gollnick H, et al. Increased vigilance in children with atopic dermatitis (AD) and/or attention deficit hyperactivity disorder (ADHD) is based on disturbed intracellular granule biology. Allergy: European Journal of Allergy and Clinical Immunology. 2014;69:242-3.* | The data published in abstract format without sufficient description and information for evaluation. The result of the Module A and Module C did not show health individual data values. | Germany |
| Yang 2013  *Yang MT, Lee WT, Liang JS, Lin YJ, Fu WM, Chen CC. Hyperactivity and Impulsivity in Children with Untreated Allergic Rhinitis: Corroborated by Rating Scale and Continuous Performance Test. Pediatrics and Neonatology. 2013((Yang M.-T.; Liang J.-S.; Chen C.-C., dtped14@pchome.com.tw) Department of Pediatrics, Far Eastern Memorial Hospital, New Taipei City, Taiwan).* | The study was to assess the attention and impulsivity control in allergic rhinitis that excluded the comorbidity with ADHD. The comparison was with the two control groups: one group did not have allergic rhinitis and ADHD and the other control group with only ADHD with no allergic rhinitis. | Taiwan |
| de Theije 2014  *de Theije CGM, Bavelaar BM, Lopes da Silva S, Korte SM, Olivier B, Garssen J, et al. Food allergy and food-based therapies in neurodevelopmental disorders. Pediatric allergy and immunology : official publication of the European Society of Pediatric Allergy and Immunology. 2014;25(3):218-26.* | It is a systematic review article and not an original study | Netherlands |
