# Supplementary material for: Allergic diseases in children with attention deficit hyperactivity disorder: a systematic review and meta-analysis
Source: BMC Psychiatry. 2017 Mar 31;17:120. doi: 10.1186/s12888-017-1281-7 (PMC5374627; doi:10.1186/s12888-017-1281-7)

**Additional file 4: Risk of bias assessment within studies**

| Risk of bias assessment for each study using Risk of Bias Assessment Tool for Nonrandomized Studies (RoBANS) tool and using additional reference guideline form A Cochrane Risk of Bias Assessment Tool: for Non-Randomized Studies of Interventions (ACROBAT-NRSI) for supporting judgments. | | |
| --- | --- | --- |
| **1. Chen 2012** [33]  **Study: retrospective case-control study** | | |
| Bias | **Authors' judgement** | **Support for judgement** |
| Selection of participants | Unclear | AHDH patients were randomly selected form the National Health Insurance Research Database (NHIRD). The age- and gender-matched control group was randomly identified from the subjects after eliminating patients who had been given a diagnosis of ADHD. Unclear to whether The potential cases of ADHD and Tics in control might be neglected for selection. |
| Confounding variables | Low | Multiple logistic regressions were performed to calculate the OR with 95% CI after adjusting for age, gender, residence location, and comorbid psychiatric disorders. |
| Measurement of exposure | Low | Those identified as having ‘attention deficit hyperactivity disorder’ (ICD-9-CM code: 314) by pediatricians, psychiatrists, and neurologists that were included as the study group |
| Blinding of outcome assessments | Unclear | Data were retrieved from national health insurance records, and most likely participants and doctors were not aware of this study. It was not clear to whether there are any personal identifiers from the database system that could influence the selection of participants. |
| Incomplete outcome data | Low | All data could from national insurance medical records and treatment information was available for analysis. |
| Selective outcome reporting | Unclear | All intended outcomes were analyses and reported but they did not clarify how they excluded those possible cases of ADHD or Tic. |
| Overall bias judgment | **Unclear risk of bias** | |

| **2. Hak 2013** [34]  **Study: retrospective nested case-control** | | |
| --- | --- | --- |
| Bias | **Authors' judgement** | **Support for judgement** |
| Selection of participants | Unclear | The study utilized data from UK General Practice Research Database (GPRD), a nationally representative database in the UK. Participants were selected based on past medical records. Cases were defined as boys with medication treatment for ADHD. Controls were male patients with no diagnosis of ADHD or treatment with ADHD drug but the potential cases might not be included. |
| Confounding variables | Low | The study included data for male patients only. Matching was done to each case on age and general practice. In addition, information on presence of low birth weight and preterm delivery were obtained as potential confounders. |
| Measurement of exposure | Low | ADHD cases were included based on the criteria of having at least one prescription of ADHD drug, within 12 months after the date of their first diagnosis. |
| Blinding of outcome assessments | Unclear | Blinding was not mentioned but since this study uses data from a medical database, there are low chances that outcomes will be influenced by study participants but not clear to whether outcome assessors were also the ones who selected the participants. |
| Incomplete outcome data | Low | Data of intended outcomes were obtained for all case (n=884) and control (n= 3536), without missing data. |
| Selective outcome reporting | Unclear | Outcome measures were analyzed and reported in accordance with the study objective but not clear whether the cases of those allergy diseases occurred before ADHD prescription were not reported. |
| Overall bias judgment | **Unclear risk of bias** | |

| **3. Kwon 2014** [36]  **Study: cross-sectional** | | |
| --- | --- | --- |
| Bias | **Authors' judgement** | **Support for judgement** |
| Selection of participants | Unclear | Children were recruited from 22 elementary schools located in seven cities in Korea. Not clear whether the school had same standard for student admission. Did not mention where (e.g. school or hospital) the ADHD diagnosis took place. |
| Confounding variables | Low | Variables such as gender, age, grade, past records of disease and developmental characteristics were enumerated. Logistic regression analysis was performed |
| Measurement of exposure | Unclear | ADHD was diagnosed according to the DSM-IV criteria, based on interviews with child psychiatrist. In addition, epidemiological questionnaires completed by the parents or caretakers were also used. |
| Blinding of outcome assessments | Unclear | Blinding was not performed. It was not clear if there were independent researcher distribute the questionnaire or the teachers at school distributed the questionnaire. |
| Incomplete outcome data | Low | Total response rate was 75%. Questionnaire were distributed to and completed by children's parents, and was collected back at schools. |
| Selective outcome reporting | High | The social and demographic of the children where not reported by either regions of the sample they collected or by each 22 schools. Only the sex and age in a pool result. |
| Overall bias judgment | **High risk of bias** | |

| **4. Romamos 2010** [37]  **Study: retrospective cross-sectional** | | |
| --- | --- | --- |
| Bias | **Authors’ judgement** | **Support for judgement** |
| Selection of participants | Unclear | ADHD children were chosen from those who participated in the German Health Interview and Examination Survey for Children and Adolescents with information on sociodemogrphics and health related topics database. Unclear to whether The potential cases of ADHD might be neglected for selection. |
| Confounding variables | Low | All confounders adjusted by logistic regression models. Testing nested models performed by using the likelihood ratio test. Missing data were included in the final model. |
| Measurement of exposure | Low | Data from KIGGS were from self-administered questionnaires and standardized parental interview conducted by trained interviewers. Those with life time diagnosis of ADHD and atopic eczema, atopic disorder cases met on the basis of medical examination and psychological diagnosis according to the ICD-10 criteria |
| Blinding of outcome assessments | Low | The study was performed in accordance with Declaration of Helsinki protocol by difference group of researchers and all personal identifiers had been removed from the database prior to this research. |
| Incomplete outcome data | Low | Outcome data that was intended for analysis were completed and missing data were considered as confounder and confounder were included their analysis. |
| Selective outcome reporting | Low | All reported results correspond to intended outcome analysis. |
| Overall bias judgment | **Unclear risk of bias** | |

| **5. Suwan 2011** [35]  **Study: case-control** | | |
| --- | --- | --- |
| Bias | **Authors' judgement** | **Support for judgement** |
| Selection of participants | Low | The participants were recruited in the Vajira Hospital, Bangkok, Thailand. ADHD children diagnosed by developmental and behavioral pediatricians, and the non-ADHD group was recruited form outpatients from well baby and dental clinics and from the department for minor surgical procedures at the same period of time. |
| Confounding variables | Low | There are expected confounders by the case-control design but the main confounders such as sex and age, within 12 months, were matched. |
| Measurement of exposure | Low | ADHD were clinically diagnosed in accordance to DSM-IV edition criteria. |
| Blinding of outcome assessments | Unclear | No blinding was performed but clinical skin prick test was unlikely to be influenced by knowledge of participants. It is not clear if the data assessor also diagnosis the allergy diseases or performed the allergy examination. |
| Incomplete outcome data | Low | All participants competed the test and no data were missing from this study |
| Selective outcome reporting | Low | All outcome measure and analyses were consistent to intended outcomes and followed the medical standardized examination protocol. |
| Overall bias judgment | **Unclear risk of bias** | |


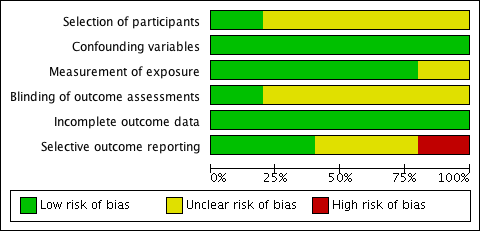

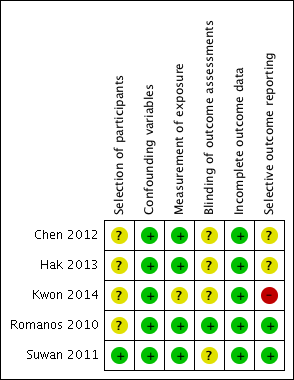

Supplement: Supplementary file 4 — Risk of bias assessment within studies. (DOCX 134 kb) [file 12888_2017_1281_MOESM4_ESM.docx]
