# Supplementary material for: Allergic diseases in children with attention deficit hyperactivity disorder: a systematic review and meta-analysis
Source: BMC Psychiatry. 2017 Mar 31;17:120. doi: 10.1186/s12888-017-1281-7 (PMC5374627; doi:10.1186/s12888-017-1281-7)
Supplement: Supplementary file 5 — Summary of results on allergic diseases in children with ADHD. (DOCX 22 kb) [file 12888_2017_1281_MOESM5_ESM.docx]

**Additional file 5: Summary of results on allergic diseases in children with ADHD**

| A summary of adjusted and unadjusted odds ratio reported in the included studies of allergic diseases association to children with ADHD | | | | | | | | | | |
| --- | --- | --- | --- | --- | --- | --- | --- | --- | --- | --- |
| **Study ID** | **Allergic diseases** | | | | | | | | | |
|  | **Asthma** | **Allergic rhinitis** | **Atopic dermatitis/ (eczema)** | **Allergic conjunctivitis** | **Any atopic disorder** | **Food allergy** | **Cow’s milk intolerance** | **Drug allergy** | **Urticaria** | **Unspecific allergies** |
| **Chen 2013** [33] | *OR 2.05 (1.90 to 2.22) | *OR 2.20 (2.06 to 2.35) | *OR 1.94 (1.75 to 2.16) | *OR 2.08 (1.96 to 2.21) |  |  |  |  |  |  |
| **Hak 2013** [34] | aOR 1.6 (1.3 to 1.9) | aOR 1.1 (0.9 to 1.4) | aOR 1.3 (0.9 to 1.7) |  | aOR 1.6 (1.3 to 1.8) | aOR 1.6 (0.7 to 3.9) | aOR 2.1 (1.1 to 4.1) |  |  | aOR 1.5 (0.8 to 1.3) |
| **Kwon 2014** [36] | aOR 1.598 (1.301 to 1.964) | aOR 1.375 (1.124 to 1.681) | aOR 1.058 (0.864 to 1.296) | aOR 0.97 (0.779 to 1.211) |  | aOR 0.936 (0.695 to 1.260) |  | aOR 1.147 (0.624 to 2.111) |  |  |
| **Romanos 2010** [37] | aOR 1.23 (0.87 to 1.72) | aOR 0.82 (0.63 to 1.06) | aOR 1.54 (1.24 to 1.93) |  |  |  |  |  |  |  |
| **Suwan 2011** [35] | aOR 1.37 (0.35 to 5.33) | aOR 3.80 (1.41 to 10.26) | aOR 1.18 (0.27 to 5.03) | aOR 3.01 (0.81 to 11.27) |  | aOR 0.93 (0.14 to 6.34) |  |  | aOR 0.22 (0.03 to 2.10) |  |
| aOR: adjusted odds ratio  *Unadjusted odds ratio (using 2 by 2 factor analysis) | | | | | | | | | | |
