# Supplementary material for: Allergic diseases in children with attention deficit hyperactivity disorder: a systematic review and meta-analysis
Source: BMC Psychiatry. 2017 Mar 31;17:120. doi: 10.1186/s12888-017-1281-7 (PMC5374627; doi:10.1186/s12888-017-1281-7)
Supplement: Supplementary file 6 — Summary of evidence quality based on GRADE approach. (DOCX 130 kb) [file 12888_2017_1281_MOESM6_ESM.docx]

**Additional file 6: Summary of evidence quality based on GRADE approach**

| No. of Participants  (No. of studies) | Risk of bias | Inconsistency | Indirectness | Imprecision | Publication bias | Quality | Relative effect (95% CI) | Anticipated absolute effects | |
| --- | --- | --- | --- | --- | --- | --- | --- | --- | --- |
|  |  |  |  |  |  |  |  | Risk without ADHD | Risk difference with ADHD |
| **Asthma** | | | | | | | | | |
| 59646  (5 studies) | Serious  1 | No serious | No serious | No serious | None  8 | Low | OR 1.80  (1.57 to 2.07) | 125 per 1000 | 80 more per 1000  (58 more to 103 more) |
| **Asthma – Nationwide studies** | | | | | | | | | |
| 51033  (2 studies) | Serious  2 | No serious | No serious | No serious | None  8 | Very low | OR 1.96  (1.67 to 2.30) | 71 per 1000 | 59 more per 1000  (42 more to 78 more) |
| **Asthma – Institutional-based studies** | | | | | | | | | |
| 8613  (3 studies) | Serious  3 | No serious | No serious | Not serious | None  8 | Very low | OR 1.67  (1.48 to 1.88) | 241 per 1000 | 106 more per 1000  (79 more to 133 more) |
| **Allergic rhinitis** | | | | | | | | | |
| 59646  (5 studies) | Serious  1 | Serious  4 | Not serious | Serious  5 | None  8 | Very low | OR 1.59  (1.13 to 2.23) | 153 per 1000 | 70 more per 1000  (16 more to 134 more) |
| **Allergic rhinitis –Nationwide studies** | | | | | | | | | |
| 51033  (2 studies) | Serious  2 | Serious  4 | Not serious | Serious  5 | None  8 | Very low | OR 1.67  (0.96 to 2.93) | 134 per 1000 | 71 more per 1000  (5 fewer to 178 more) |
| **Allergic rhinitis – Institutional-based studies** | | | | | | | | | |
| 8613  (3 studies) | Serious  3 | Serious  4 | Not serious | Serious  5 | None  8 | Very low | OR 1.48  (1.00 to 2.17) | 325 per 1000 | 91 more per 1000  (0 fewer to 186 more) |
| **Atopic dermatitis** | | | | | | | | | |
| 59646  (5 studies) | Serious  1 | Serious  4 | Not serious | Not serious | None  8 | Very low | OR 1.43  (1.09 to 1.88) | 100 per 1000 | 37 more per 1000  (8 more to 73 more) |
| **Atopic dermatitis - Nationwide studies** | | | | | | | | | |
| 51033  (2 studies) | Serious  2 | Serious  4 | Not serious | Not serious | None  8 | Very low | OR 1.74  (1.36 to 2.22) | 94 per 1000 | 59 more per 1000  (30 more to 94 more) |
| **Atopic dermatitis – Institutional-based studies** | | | | | | | | | |
| 8613  (3 studies) | Serious  3 | Not serious | Not serious | Not serious | None  8 | Very low | OR 1.16  (1.00 to 1.35) | 100 per 1000 | 14 more per 1000  (0 fewer to 30 more) |
| **Allergic Conjunctivitis** | | | | | | | | | |
| 41908  (3 studies) | Serious  3 | Serious  4 | not serious | Serious  5 | None  8 | Very low | OR 1.69  (1.04 to 2.76) | 203 per 1000 | 98 more per 1000  (6 more to 210 more) |
| **Allergic Conjunctivitis - Nationwide studies** | | | | | | | | | |
| 37715  (1 study) | Serious  6 | Not serious | Not serious | Not serious | None  8 | Very low | OR 2.08  (1.96 to 2.21) | 203 per 1000 | 143 more per 1000  (130 more to 157 more) |
| **Allergic Conjunctivitis – Institutional-based studies** | | | | | | | | | |
| 4193  (2 studies) | Serious  7 | Serious  4 | Not serious | Serious  5 | None  8 | Very low | OR 1.36  (0.79 to 2.36) | 175 per 1000 | 49 more per 1000  (32 fewer to 159 more) |
| **Food allergy** | | | | | | | | | |
| 8613  (3 studies) | Serious  3 | Not serious | Not serious | Serious  5 | None  8 | Very low | OR 1.13  (0.88 to 1.47) | 75 per 1000 | 9 more per 1000  (8 fewer to 31 more) |
| *The corresponding odds (and its 95% confidence interval) is based on the assumed odds in the comparison group and the relative effect of the exposure (and its 95% CI).  **CI**: Confidence interval; **OR**: Odds ratio | | | | | | | | | |
| Criteria provided by GRADE handbook:  **Very serious limitations:** crucial limitation for one or more criteria sufficient to substantially lower confidence in the estimate of effect.  **Serious limitations:** potential limitations are likely to lower confidence in the estimate of effect.  **No serious limitations:** no apparent limitation. | | | | | | | | | |
| 1. Four of the studies had limitation on the selection of participants and blinding of outcomes assessments by their study designs but one study had unclear risk of bias in the measurement of exposure and one study indicated high risk of bias on the outcome reporting which lowered the quality of the observational evidence. 2. The proportion of information was from two studies indicated with limitation on selection of participants and blinding by their study designs but the unclear risk of selective reporting which lowered the quality of the observational evidence. 3. The proportion of information was from two studies indicated limitation on selection of participants and blinding of outcome assessments by their study designs but one study had high risk of outcome reporting, which lowered the quality of the observational evidence. 4. There is an indication of significant inconsistency (I^2^>80%) 5. Information were from high heterogeneity and small sample size with a wide confidence interval 6. The information is based on one study, which had limitation on the selection of participants, blinding of outcomes assessments and selective outcome reporting by the study design. 7. The information from two studies that had limitation on selection of participants, measurement of exposure, blinding of outcome assessment by their study designs but one with high risk of bias on selective outcome reporting which lowered the quality of the observational evidence. 8. The possibility of publication bias is not disregarded but it was not considered to downgrade the quality of the observational evidence. | | | | | | | | | |
